# Acute Hydrocephalus, or Water in the Head, an Inflammatory Disease, and Curable Equally and by the Same Means with Other Diseases of Inflammation

**Published:** 1841-01

**Authors:** 


### Art. VII.

*Acute Hydrocephalus, or Water in the Head, an Inflammatory Disease, and curable equally and by the same means with other diseases of Inflammation.* By David D. Davis, m.d., m.r.s.l.; Professor of Obstetric Medicine in University College, and one of the Physicians to University College Hospital.—*London*, 1840. 8vo.

When we first glanced at the title of Dr. Davis's work, we suspected that he had chosen a subject too fully investigated by preceding authors to leave much room for fresh discoveries,—at the present time. Whether any improvement in the mode of treating the disease had occurred to the writer, was a question which we hoped to have the opportunity of answering in the affirmative. An attentive and impartial examination of the volume has shown us that our suspicion was correct; for we have failed to discover in it the smallest addition to our pathological knowledge. Nor can we award higher praise to the treatment advocated, which can scarcely be considered novel, even in its details. The literary character of the work is even still more defective, and can hardly be regarded by the most lenient judgment as creditable either to the author or the celebrated school to which he belongs. No doubt the extremely faulty style in which a good deal of the work is written, must be attributed to mere carelessness on the part of the author, as it is impossible that the manifold blunders with which it abounds, can be the result of ignorance. And even on this assumption we cannot acquit the writer of blame of no light kind, more especially in his relation as a teacher of medicine. If his avocations prevented him from paying that attention to the composition and editing of his book which every book ought to receive, he might surely have obtained the assistance of some of his literary friends of greater leisure than himself; and we venture to affirm that he could have found many pupils in his own college, the revision of it by any one of whom would have saved his reputation from the literary discredit which, in the judgment of persons ignorant of his real powers, must attach to it after the present publication.

There is another unfortunate character of composition observable in several parts of Dr. Davis's book, however, for which the most lenient interpretation must hold him exclusively responsible, as it is the result of evident intention on the part of the author: this is the fashioning his style to meet the comprehension and views of the "general" reader. Our pages bear ample testimony of our desire that the public should be instructed in everything relative to the preservation of health and the prevention of disease; and we conceive that the medical philosopher can aspire to no higher or more dignified office than that of such an instructor. But we must ever deprecate the attempt in works on scientific or practical subjects, designed for the profession, to write down to the standard of non-professional readers. Whether intended or not, the effect of this is—when it has any effect, although we suspect the results are generally of a negative kind—not so much to instruct such persons in matters which they can understand, and which may be useful to them, as to turn their thoughts towards the doctor who writes so incomprehensibly-learnedly, and who, doubtless, can heal so incomparably-cleverly.

If our limits will allow, we may, before concluding, extract a few passages from Dr. Davis's book in illustration of our meaning, and in justification of our censure. For the present we turn to the more agreeable task of examining the strictly professional character of the publication.

The object of the author, as stated in his preface, is to prove that acute hydrocephalus is an iuflammatory disease of the vessels and investing membranes of the brain, and that it calls for the same remedies as other inflammations. The accuracy of this doctrine is inferred from the disorganization of the blood common to this and to all inflammatory disorders, and evinced by the following morbid appearances: 1, opacities and fibrinous adhesions of the contiguous membranes of the brain; 2, fibrinous coatings, linings, and deposits on the surface of the organ, and within its cavities; 3, serous effusions, both on the cerebral surface and between the convolutions, and also between the membranes; 4, occasional purulent formations, with disorder of tissue, as of the temporal bone, including the organ of hearing; 5, the presence of a peculiar fluid in the ventricles, infiltrated from the blood by the inflamed cerebral tissues, and which Berzelius regards as serum of the blood, diluted with seven times its volume of water.

The various theories of acute hydrocephalus are historically sketched in the Introduction, and this we deem the best part of Dr. Davis's work; although even this is marked by some striking defects. The disease was first described as a distinct affection by Mr. Pasley, of Glasgow, who records a case of it in the third volume of the Edinburgh Medical Essays. In 1768 it was the subject of a monograph by Dr. Robert Whytt, Professor of Medicine at Edinburgh, and also gave occasion to several papers by Drs. Fothergill and Watson in the fourth volume of the Medical Observations and Enquiries. In the sixth volume of the same work is a case of the disease cured by mercury, published by Dr. Dobson, of Liverpool, in 1775. Up to that period the current theory was, that hydrocephalus resulted from effusion into the ventricles, consequent on a a laxity of the exhalents, or on a watery state of the blood; in short, that it was an acute idiopathic dropsy. In opposition to this theory, Dr. C. Quin published an inaugural dissertation in 1779, in which he maintained that hydrocephalus is inflammatory, and consists in plethora of the vascular tissue of the brain, resulting in the effusion of an aqueous fluid, and demanding an antiphlogistic treatment. This opinion was expressed by Dr. Withering in his account of digitalis, published in 1785; and also by Dr. Rush, of Philadelphia, in an essay published in 1793; by Dr. Patterson, of Londonderry, in a series of letters to Dr. Quin, printed at Dublin in the following year; and by Dr. Garnet, in a short essay contained in the fifth volume of the London Medical and Physical Journal for 1801. The doctrine was combated, though not the treatment, in the essay of Dr. J. Cheyne in 1818. In 1814 Dr. Carmichael Smyth endeavoured to revive Whytt's theory of effusion from debility and relaxation of vessels, and expressed a conviction that "neither the brain itself nor its membranes in any case of genuine hydrocephalus that ever yet has been examined, showed any appearance of inflammation, or of the usual effects of this having taken place." Professor Monro, of Edinburgh, still holds the opinion of Smyth. The next writer on hydrocephalus was Dr. Yeats, who addressed, in 1815, a letter to Dr. Wall, Clinical Professor at Oxford, urging attention to the premonitory symptoms, which he says are easily controlled; but if neglected, lay the foundation for the disease. Some time after appeared the highly practical and valuable work of Dr. Gölis, of Vienna, translated by the late Dr. Gooch. Of this work Dr. Davis tells us he intends, in the ensuing pages, to point out the superiority to other treatises, and to supply any practical deficiencies.

We expected that some notice would have been taken of the labours of some of those French pathologists who support the same side of the question as our author; for instance, Martinet, Parent-Duchatelet, and Lallemand. The omission is inexcusable in this age of pathological research. Some mention, too, should have been made of that curious discovery of Magendie and Guillot—the hygrometric property of the brain. The first of these physiologists pointed out that, to show the cerebro-spinal ventricular fluid, the animal must be examined immediately after death, otherwise the fluid is absorbed, and disappears in the midst of the nervous substance. Guillot has shown, first, that during life, and in health, the ventricles of the brain are full of serosity; that the quantity of this fluid decreases in proportion to the interval between death and the dissection; that the fluid so disappearing is to be found in the cerebral substance; that the brain is hygrometric, so that a piece of the cerebrum of a dog just killed, and plunged into water or serum, absorbs its own weight of these fluids; and that differences exist in different animals, and in the same animals, according to age and the interval that has occurred between its death and the period of examination. A new light is cast on the subject of cerebral effusion by this discovery, and henc forward it will be necessary, not merely to estimate the quantity of fluid found in the ventricles or cells of the subarachnoid cellular tissue, but likewise of that contained in the substance of the brain itself. If this organ is soft, and yields on pressure a large quantity of fluid, we are warranted in concluding that the effusion is dropsical. This is seldom seen, however, in acute hydrocephalus; and, when it is, it must be regarded as accidental.

Within the last fifteen or twenty years a doctrine has sprung up in the French school, which attributes acute hydrocephalus to deposition of tubercular granulations on the under surface of the cerebral layer of the arachnoid membrane; in other words, maintaining the strumous nature of the disease. MM. Guersent, Gherard, Rufz, Piet, Bequerel, and Dr. Hennis Greene, have zealously supported this view, and have accumulated a vast mass of evidence in its support.

Dr. Davis passes over the distinction of external and internal hydrocephalus, and the several forms of each, and restricts himself to that form of the latter in which the effused fluid is contained in the ventricles of the brain. He divides this into three stages: 1, the formative (or predisposing); 2, the inflammatory; and 3, that of effusion, which is commonly fatal.

The first or formative stage of hydrocephalus is more diffusely described than is compatible with clearness. We do not think it necessary to give even an abstract of the description, which varies in no important respect from what is found in every work of character on the subject. We were struck with the omission of one symptom, namely, alternate evening febrile exacerbations and remissions, constituting "Infantile Remittent Fever," which is certainly a highly important premonitory symptom of the disease.

Minutely as the second or inflammatory stage is described, yet no mention is made of the contraction of the pupil at the commencement, which marks the highly irritable retina; nor yet of the change from contraction to dilatation, so uniformly observed when effusion is taking place. Now this state of the pupil is as important a sign as the slow, unequal, and intermitting, "and easily quickened pulse, which indicates effusion ...." "*An eruption*" on the shoulders and nape of the neck is said by Dr. Davis to be symptomatic of this and the third stage of the disease; but of its specific character nothing is said.

The symptoms of the third stage are accurately related, allowance being made for obscurity and other vices of style, which in this part of the book seem to have attained their highest pitch. The general description of symptoms would have been more complete if some notice had been taken of their chief varieties, which are sometimes a puzzle to the medical attendant. Thus, the premonitory stage may be absent, and the attack be *suddenly* ushered in by strong convulsions. Sometimes the pulse is never slow, or never presents that change from slowness to extreme frequency, which is so characteristic of the last stage. Occasionally there is neither permanent contraction, nor dilatation of the pupil, but an irregularity in the contraction of the iris; so that when a candle is brought near it will dilate instead of contracting, and when the candle is withdrawn, it will contract instead of dilating. Again, children have been seen to retain their consciousness to the last.

The chapter on the diagnosis of acute hydrocephalus, under the head of pathognomonic symptoms, is a mere abbreviation of the previous one on symptoms. It offers no clue to the more exact discrimination of this from the other maladies for which it may be mistaken as softening, arachnitis of the base of the brain, and of the ventricles (Martinet and Parent-Duchatelet), inflammation and ulceration of the ileum and of the other small intestines, and intestinal worms. The subject of diagnosis is in point of fact absolutely overlooked: a striking proof of the negligent and hasty construction of this work, notwithstanding the self-complacency with which the author speaks of "the above *accurate and carefully drawn* picture," alluding to his diagnostic sketch.

In treating of the specially predisposing causes "to" (of) acute hydrocephalus, great stress is very justly laid on the irritation of teething, though there is nothing new in this to justify the phrase, "it may be here advantageously *intimated* to the reader that the irritation from teething is in nine out of ten cases, the occasional cause of acute hydrocephalus during the three first years of life." Another frequent predisposing cause is the too common substitution of artificial for the natural food, during the early period of infancy, a practice justly condemned by the author. A curious example of the predisposing power of mental emotions of the mother during the close of pregnancy, is extracted from the work of Dr. Gölis, who relates that in 1809 most of the children born after the bombardment of Vienna, were seized with convulsions, twenty or thirty days after birth, and died. Traces of inflammation were found within the cranium, and in the ventricles effusions of lymph and serum. The other predisposing causes enumerated, do not call for any observations.

Dr. Davis devotes a large portion of his volume to the examination of the proximate cause of hydrocephalus. He tells us that collections of serous fluid in the cerebral cavities were seen and are recorded by nosologists prior to the last century, but were attributed by none to inflammatory action. A dissection of this kind was given in 1676 by Borelli, in his Histories and Medico-Physiological Observations (obs. 38); another by Morgagni, in his great work De Sedibus et Causis Morborum, (ep. 1, act. 2;) and others again in the 6th, 7th, and 8th ep. of the 1st book. We are warranted in concluding from the silence of writers at this period, that the inflammatory origin of these effusions was unknown, and indeed until the appearance of Dr. Quin's dissertation in 1779, upwards of a century after, no new light was thrown on the subject. This author is undoubtedly the parent of the inflammatory theory, though from his father he learnt the distinction of hydrocephalus into acute and chronic. The inflammatory nature of the acute variety, he inferred from observing that when the headach was severest, vascular excitement was strongest, and that the superficial vessels of the head evinced a plethora, further shown by repeated epistaxis before the attacks. The vessels of the cerebrum and cerebellum too were found by him much injected; and opacities, thickening, patches of coagulable lymph, and serous effusions in the ventricles, were observed in several post-mortem examinations. It is singular that neither Dr. Quin nor any of his contemporaries adopted a line of treatment in accordance with this doctrine. Indeed, it is only of late years, that the profession has treated hydrocephalus on the antiphlogistic plan; the contrary doctrine inculcated by Dr. Carmichael Smyth having had great weight in retarding the progress of the new theory. The works of Drs. Underwood, John Clarke, and Abercrombie, corroborate the modern view, yet Dr. Davis, never naming the two latter authors, questions if it is generally received, and says, "but unfortunately, this pathology has been more of a tacitly admitted principle, which, however, has not been unfrequently denied, than a doctrine of an *operative living faith*," (p. 211.) A series of cases with dissections from the admirable work of Gölis, are introduced to show that an inflammatory state of the blood-vessels of the encephalon is the true proximate cause of hydrocephalus; and these are succeeded by several other dissections, published by Dr. Cheyne shortly before the date of Gölis's work.

Here Dr. Davis closes his argument for the inflammatory nature of acute hydrocephalus; and though we agree in much that he has said, and admit the weight of the concurrent testimony he has adduced, yet we cannot award him the praise of having shed any new light on the pathology of hydrocephalus. Neither in his arguments nor in his pathological researches, do we discover any of that cogency or originality which we were led to expect.

*Treatment.* The most important of the remedies of hydrocephalus is, in Dr. Davis's opinion, *bloodletting;* which may be employed as a prophylactic in the premonitory stage, when the only symptoms are cerebral vascular turgescence with pyrexia. The first bleeding, to be effectual, should be carried to complete fainting, the blood being drawn quickly from a large orifice. A repetition of the depletion will rarely be necessary, as the disease will generally have been arrested by this vigorous treatment. For a child below five years of age, Dr. Davis considers cupping behind the ears preferable to venesection, on account of the diminutiveness of the veins at the bend of the elbow, and to leeches, on account of the difficulty of computing the exact quantity of blood abstracted by them. The extent to which cupping may be carried is thus laid down: From an infant a year old, from 4 to 5 oz. of blood may be abstracted, and half an ounce or an ounce more on the second day of stupor, especially if the head is hot; from one two years old, from $5\frac{1}{2}$ to 6 oz.; from one of from 3 to 5 years of age, from 5 to 10 oz.; from one of from 6 to 10 years of age, from 10 to 18 oz. But in all these cases, fainting should be produced. Next to cupping, the opening of the jugular vein is most recommended.—Agreeing generally with this plan of treatment in decidedly acute cases in children of ordinary powers, we must caution our younger readers against its indiscriminate employment. There are not a few cases, even of the inflammatory kind, in which it is only applicable in a most modified degree; and there are many cases of hydrocephalus in which it is altogether inadmissible.

As soon as the child has pretty well recovered from the faintness induced by the bleeding, an emetic should be given; for instance, $\frac{1}{4}$ gr. of tartar emetic with 5 grs. of ipecacuanha, and vomiting promoted by draughts of fluid at intervals. In this way the morbid excitability of the heart and arteries is powerfully subdued. The sickness and nausea may continue for several hours with ultimate advantage, a fact which induces Dr. Davis to add this piece of encouragement by way of caution, "Whilst on this subject, it may not be improper to caution *parents* against placing any confidence in certain alarming, and otherwise uncalled for observations of *druggists' apprentices, and other young gentlemen occupied in shops of pharmacy*, about the magnitude of doses of medicines intended for young children."

Next to bloodletting and emetics, the author places the reduction of temperature, by means of cold applications; for instance, "iced water, or other *solutions of water* impregnated with a variety of salts, vinegar, &c.," and contained in a Mackintosh's water-cushion, which should be changed every half hour, as long as it appears agreeable to the child. In the incipient stage of the disease, if the naked hand is applied to the uncovered forehead or occiput, a marked excess of temperature will be noticed, and more accurately still, by a pocket thermometer. As long as the head continues preternaturally hot, so long will the infant, if conscious, appear to hail the cushion, and so long ought it to be continued.

To blisters, the author does not assign a high place among the remedies of acute hydrocephalus; he believes them absolutely injurious when used as a first remedy, without previous ample bloodletting. He recommends that they should be applied over a large surface of the parietal regions, the forehead and occiput being constantly bathed with evaporating lotions.

Mercury he considers a valuable remedy in this disease; as a purgative he gives it in the form of calomel in doses of 2 or 3 grs., sometimes twice that quantity, with 6, 8, or 12 grs. of jalap, according to the patient's age, as soon as the sickness consequent on the emetic, shall have ceased. Where the bowels are obstinately costive, he adds occasionally $g^{tt.}\frac{1}{4}$ to $g^{tt.}\frac{1}{2}$, or even more, of croton oil. Calomel is no less valuable as a cholagogue, when the secretion of the liver is suspended, or in other words, its circulation much obstructed; which frequently predisposes to affections of the brain, by destroying the balance of the circulation, so that congestion of the cerebral vessels takes place. When exhibited with a view to promote the biliary secretion, Dr. Davis advises the dose to be from 1 to 3 grs. every three hours. Frequently a very active purgative will be required at the same time. A third object in employing calomel is to produce its specific effect, with the view of subduing inflammatory action. On this, however, the author tells us he does not much depend, except the system shall have been reduced by the lancet. At the same time he would administer calomel in cases where bleeding would be too late, or otherwise inadmissible. On the whole, he considers mercury inferior as a curative agent to any of the other active remedies enumerated, and this opinion derives confirmation from the results of Dr. Cheyne's treatment of twenty cases of hydrocephalus by bleeding and calomel. The number of recoveries was only nine.

Such is Dr. Davis's account of the treatment of acute hydrocephalus. A series of paragraphs, thirteen in number, succeeds, detailing the "circumstances indicative of danger of acute hydrocephalus, which should operate as a warning to *parents and guardians* to take the advice of their medical friends in good time." We cannot see the use of this superfluous recapitulation. If there are so many indications of liability to the disease, parents and guardians will be bewildered by the mere enumeration, even if they could be supposed competent to appreciate the indications and the succeeding cases which are meant as illustrations.

The work closes with the details of six cases, successfully treated by the author at University College Hospital, on the rigorous plan already described. We are told that the records of the institution present similar results in the vast majority of cases, and are assured that no less success may be expected from the treatment in private practice, so long as the disorder is submitted early enough to the notice of the practitioner.

In thus closing our sketch of the author's treatment of hydrocephalus, we cannot help repeating that it has no claims either to novelty or originality; scarcely a modern author or practitioner of reputation but professes similar therapeutic principles. If the reader will but turn to Dr. John Clarke's excellent Commentary on the Diseases of Children, he will read a confirmation of our remarks in the observations on blood-letting in inflammation of the brain, and in short, on the treatment of the disease in general.

---

